# Satellite Selection Strategy and Method for Signals of Opportunity Navigation and Positioning with LEO Communication Satellites

**DOI:** 10.3390/s25010267

**Published:** 2025-01-06

**Authors:** Yanhua Tao, Yang Guo, Shaobo Wang, Chuanqiang Yu, Zimo Zhu

**Affiliations:** Laboratory of Intelligent Control, Rocket Force University of Engineering, Xi’an 710025, China

**Keywords:** GNSS-denied environment, LEO communication satellite, space-based SOPs, doppler shift (DS), satellite selection strategy, precise point positioning (PPP)

## Abstract

Experts and scholars from various nations have proposed studying low Earth orbit (LEO) satellite signals as the space-based signals of opportunity (SOPs) for navigation and positioning. This method serves as a robust alternative in environments where global navigation satellite systems (GNSS) are unavailable or compromised, providing users with high-precision, anti-interference, secure, and dependable backup navigation solutions. The rapid evolution of LEO communication constellations has spurred the development of SOPs positioning technology using LEO satellites. However, this has also led to a substantial increase in the number of LEO satellites, thereby reintroducing the traditional challenge of satellite selection. This research thoroughly examines three critical factors affecting positioning accuracy: satellite observable time, satellite elevation, and position dilution of precision (PDOP). It introduces a strategic approach for selecting satellites in LEO SOPs navigation and positioning. Simulation outcomes confirm that this satellite selection strategy effectively identifies visible satellites, ensuring precise positioning through LEO SOPs.

## 1. Introduction

Positioning technology has become an indispensable part of our daily lives with the rapid advancement of modern technology. From navigation to social media, and from logistics to agriculture, location technology is transforming the way we live and work. However, the existing Global Navigation Satellite System (GNSS) faces challenges, such as low signal power at ground level, single frequency points, susceptibility to electromagnetic interference, and high construction and maintenance costs. Therefore, researching backup navigation and positioning systems for GNSS-denied environments has become a crucial direction and challenge for various countries and regions to address navigation warfare concerns.

Signals of opportunity (SOPs) positioning is an effective backup navigation and positioning method. SOPs positioning is the technology that utilizes existing signals not originally intended for positioning purposes [1]. These signals typically originate from various commercial, civilian, or scientific sources, such as television, radio, and mobile communication networks. The primary advantage of SOPs positioning technology is its ability to leverage existing infrastructure, thereby avoiding the high costs associated with establishing a dedicated positioning system. Currently, from the perspective of signal sources, SOPs positioning encompasses Wi-Fi positioning [2], broadcast signal positioning [3], acoustic positioning technology [4], geomagnetic positioning [5], and light pattern recognition positioning [6]. From a positioning principles perspective, SOPs positioning includes time of arrival (ToA) [7], time difference of arrival (TDoA) [8], angle of arrival (AoA) [9], signal strength (SS) [10], and doppler shift (DS) positioning [11]. Particularly in recent years, the rapid development of low Earth orbit (LEO) constellations such as Iridium-NEXT [12] and Starlink [13] in the United States; Hongyan, Hongyun, and China Satellite Network in China; and OneWeb [14] in the United Kingdom has accelerated advancements in SOPs positioning technology for LEO communications satellites. In 2019, Qin H.L. et al. proposed a positioning technology based on Iridium SOPs. They obtained doppler shift (DS) information through the single tone signal of Iridium and established an instantaneous doppler positioning model. The experimental results demonstrated positioning accuracy better than 200 m [15]. In 2020, Qin H.L.’s team proposed a positioning technology based on Orbcomm satellite SOPs, achieving an accuracy better than 140 m in the final experimental results [16]. In 2022, the team completed research on a positioning method using Starlink SOPs. By analyzing the pilot signals of Starlink, they extracted the instantaneous doppler shift using the 11.325 GHz and 11.575 GHz frequency signals of Starlink. The experimental results showed a two-dimensional (2D) positioning accuracy better than 15 m with an observation time of less than 5 min [17]. In 2021, Mohamad Orabi, Joe Khalife, and Zaher M. Kassas proposed a doppler integrated navigation framework for Iridium NEXT and Orbcomm satellites. The experimental results indicated that using one Orbcomm satellite and four Iridium NEXT satellites resulted in a positioning error of 22.7 m [18]. In the same year, Zaher M. Kassas’s team published the first carrier phase tracking and positioning results with Starlink LEO satellite signals. They proposed a Starlink signal carrier phase tracking algorithm based on an adaptive Kalman filter. The experimental results clearly indicated a two-dimensional error of 7.7 m for known altitude information and two-dimensional and three-dimensional errors of 25.9 m and 33.5 m, respectively, for unknown altitude information [19]. Moreover, in 2022, the team proposed a framework for positioning with carrier phase difference low Earth orbit (CD-LEO) measurements. This framework uses a base and a rover to navigate using the Orbcomm LEO constellation without prior knowledge of the rover’s position. The impact of ionospheric and tropospheric delays on carrier phase and CD-LEO measurements was also discussed, with experiments showing a final positioning error of 11.93 m [20]. In addition to the previously discussed research on doppler frequency shift positioning technology for LEO satellite signals of opportunity, the current research also explores positioning technologies utilizing angle-of-arrival estimation and time difference of arrival for LEO satellite signals of opportunity. In 2015, Yaser Norouzi1, Elham Sadat Kashani, and Abdollah Ajorloo proposed the angle of arrival-based target localization method with the LEO satellite observer. This positioning method uses spherical trigonometry and singular value decomposition (SVD) to directly achieve target localization [21]. In 2021, Sterling Thompson, Scott Martin, and David Bevly proposed the single differenced doppler positioning method with low Earth orbit signals of opportunity and angle of arrival estimation. With the AOA estimates and differential doppler positioning, neither base stations nor mobile receivers require precise knowledge of the LEO satellite states [22]. In 2024, Antonello Florio et al. proposed coarse positioning technology through angle-of-arrival estimation of signals of opportunity based on the LEO satellite. The AOA estimation is done in real time using phase interferometry through a dedicated hardware architecture, and the final positioning accuracy demonstrates the effectiveness of the positioning technology [23]. In 2022, Barry and Weiss proposed an alternative PNT based on the time difference of arrival (TDOA) of LEO signals of opportunity. Based on the LEO signals, no a priori knowledge of the LEO orbit parameters nor the time of transmission of the signals is assumed [24].

In summary, research teams worldwide have extensively studied and analyzed the positioning technology for LEO communications satellites using signals of opportunity (SOPs). With the rapid expansion of LEO constellations, the number of satellites is increasing exponentially, posing the challenge of selecting the appropriate satellite for positioning when multiple satellites are observed simultaneously. To address this, scholars have engaged in various studies. As early as the 1980s, Kihara and Okada suggested that the influence of satellite selection on positioning accuracy could be quantified by the Geometric Dilution of Precision (GDOP), where a smaller GDOP indicates higher positioning accuracy [25]. Over the years, satellite selection based on GDOP has evolved into three main approaches: minimizing the number of GDOP calculations, recursively searching and calculating the GDOP, and treating the GDOP calculation as a constrained combinatorial optimization problem. However, most GDOP research has focused on mid- to high-orbit constellations specifically designed for navigation and positioning. In 2023, Junqi Guo, Yang Wang, and colleagues proposed a fast satellite selection algorithm for positioning in LEO constellations. This algorithm is applicable to both user terminals with limited satellite signal access and ground stations with extensive access that outperform quasi-optimal, rotation partitioning, and recursive algorithms [26]. Building on previous studies, this manuscript primarily investigates satellite selection strategies and methods for SOPs positioning using LEO communication satellites, emphasizing three critical factors affecting positioning accuracy: satellite observable time, satellite elevation, and Position Dilution of Precision (PDOP). This constitutes the main contribution and highlight of the paper. The proposed method is applicable not only to the Starlink constellation but also to other LEO constellations.

The remainder of the paper is structured as follows: Section 2 delves into the principles of positioning using (SOPs) of low Earth orbit (LEO) communication satellites. Section 2.3 introduces the concept of position dilution of precision (PDOP). Section 3 investigates the impact of satellite elevation and orbital altitude on positioning accuracy, based on studies conducted by other scholars. Section 4 proposes a satellite selection strategy for LEO communication satellites’ SOPs-based navigation and positioning, informed by the preceding analysis. Section 5 concludes with a comprehensive simulation analysis. Finally, Section 6 gives the concluding remarks.

## 2. The Principle of DS Positioning with LEO Communication Satellites

### 2.1. DS Calculation of LEO Communication Satellite Signals

Due to the asynchronous nature of LEO communication satellites, there is relative motion between users and the LEO satellite orbiting the Earth, which causes the user’s signal reception frequency to deviate from the original signal frequency, leading to the doppler effect. The extra frequency generated by this doppler effect is termed the doppler shift.

**Figure 1:**
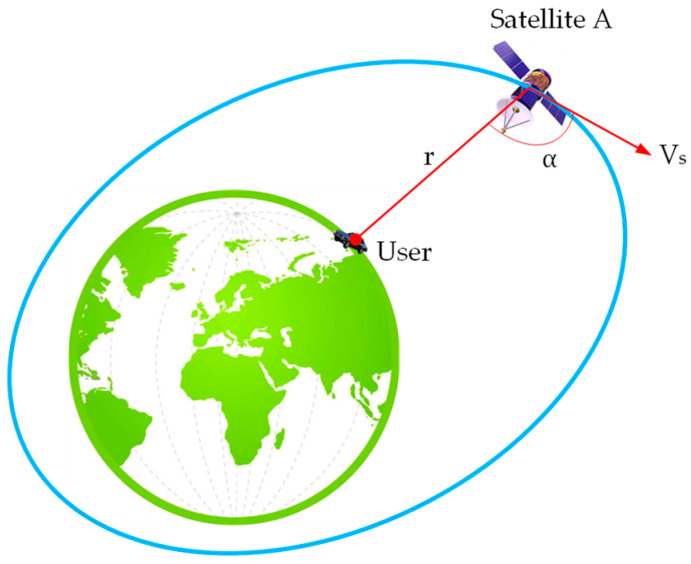
Schematic diagram of DS.

As shown in Figure 1, LEO communication satellite A orbits the Earth at a speed of *V_s_*. The signal emitted by satellite A at time t can be represented by $u_{S}=U_{t}\cos\left(\omega_{t}t+\phi_{0}\right)$, where $\omega_{t}$ is the angular frequency of the signal, and $\phi_{0}$ is the initial phase of the signal. The signal received by the user is as follows:

(1) $$u=U\cos\left[\omega_{t}\left(t-\tau \right)+\phi_{0}\right],$$

where $\tau =\frac{r}{c}$ is the signal propagation time–delay caused by the relative motion of satellite A and the user; $r=r_{0}-V_{r}t$ is the change in distance between satellite A and the user; $r_{0}$ is the initial distance between satellite A and the user; $V_{r}=V_{s}\cos\alpha$ is the radial velocity between satellite A and the user, *V_s_* is the speed of satellite A; α is the angle between *V_s_* and the user’s line of sight. Therefore, it can be calculated that the phase of the user’s received signal is as follows:

(2) $$\phi =\omega_{t}\left(t-\frac{r_{0}-V_{r}t}{c}\right)+\phi_{0},$$

For Equation (2), by taking the derivative of phase to time, the angular frequency received by the user can be obtained as follows:

(3) $$\omega_{r}=\frac{d\phi }{dt}=\omega_{t}\left(1+\frac{V_{r}}{c}\right),$$

Based on the Equations (1)–(3), it can be concluded that the instantaneous frequency received by the user is different from the signal frequency emitted by satellite A. When considering only the relative motion, its frequency difference is doppler shift as follows:

(4) $$f_{d}=2\pi \omega_{r}-2\pi \omega_{t}=f_{t}\frac{V_{r}}{c},$$

where $V_{r}=V_{s}\cos\alpha$, $f_{t}=2\pi \omega_{t}$ is the transmission frequency of the signal. From the above Equation (4), we can conclude that the magnitude of DS depends on the satellite signal frequency and relative motion velocity between satellite A and the user, and the positive or negative of DS depends on the direction of relative motion, which is determined by α.

### 2.2. The Principle of DS Positioning

**Figure 2:**
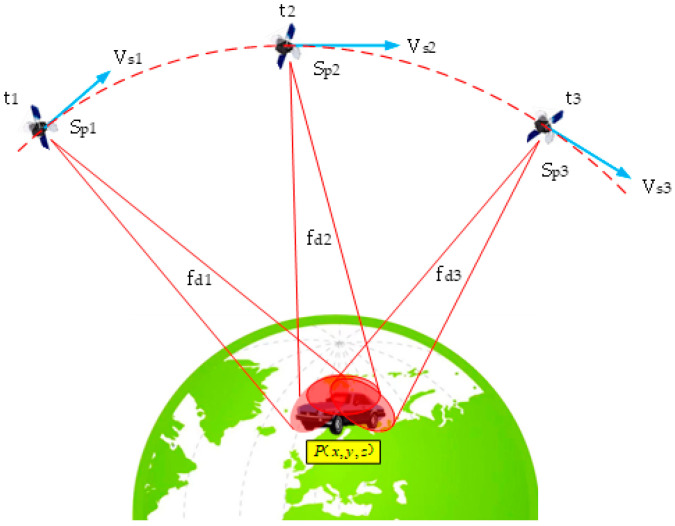
The principle of DS positioning.

Given the unique nature of DS positioning, user positioning can be accomplished by monitoring the DS variations of a single satellite at different times. Alternatively, observing the DS of multiple satellites simultaneously can also achieve user positioning. Assuming that only frequency changes due to relative motion are considered, continuous observation of the DS of satellite A is conducted, as depicted in Figure 2. The DS of the signals received by the user at times t1, t2, and t3 are fd1, fd2, and fd3, respectively, as shown in Equation (5).

(5) $$f_{di}=\frac{f}{c}\frac{V_{si}\cdot (P-S_{i})}{\left\|P-S_{i}\right\|}=\frac{f}{c}\frac{V_{six}(P_{x}-S_{ix})+V_{siy}(P_{y}-S_{iy})+V_{siz}(P_{z}-S_{iz})}{\sqrt{{\left(P_{x}-S_{ix}\right)}^{2}+{\left(P_{y}-S_{iy}\right)}^{2}+{\left(P_{z}-S_{iz}\right)}^{2}}},$$

where i=1,2,3 denotes the three positions of satellite A; f is the transmission frequency of the satellite signals; c is the speed of light; $V_{si}$ the vector velocity of satellite A; $S_{i}$ is the spatial position of the satellite; $P=(P_{x},P_{y},P_{z})$ is the position of the test point. The meanings of the other letters in Equation (5) are shown in Figure 2.

Linearize Equation (5) around the predicted position $P_{0}=(P_{x0},P_{y0},P_{z0})$ to derive Equation (6), as follows:

(6) $$\begin{array}{cc} f_{di}(S_{ix},S_{iy},S_{iz}) & =f_{di0}(P_{x0},P_{y0},P_{z0})+\frac{\partial f_{di}}{\partial P_{x}}(P_{x}-P_{x0})+\frac{\partial f_{di}}{\partial P_{y}}(P_{y}-P_{y0})+\frac{\partial f_{di}}{\partial P_{z}}(P_{z}-P_{z0})+h.o.t\\ & =f_{di0}(P_{x0},P_{y0},P_{z0})+\frac{f}{c}\left\{\begin{array}{l} \left[\frac{V_{six}}{d_{i}}-\frac{V_{si}{\left(P_{0}-S_{i}\right)}^{T}(P_{x0}-S_{ix})}{d_{i}^{3}}\right](P_{x}-P_{x0})\\ +\left[\frac{V_{siy}}{d_{i}}-\frac{V_{si}{\left(P_{0}-S_{i}\right)}^{T}(P_{y0}-S_{iy})}{d_{i}^{3}}\right](P_{y}-P_{y0})\\ +\left[\frac{V_{siz}}{d_{i}}-\frac{V_{si}{\left(P_{0}-S_{i}\right)}^{T}(P_{z0}-S_{iz})}{d_{i}^{3}}\right](P_{z}-P_{z0}) \end{array}\right\}+h.o.t \end{array},$$

where $d_{i}=\sqrt{{\left(P_{x0}-S_{ix}\right)}^{2}+{\left(P_{y0}-S_{iy}\right)}^{2}+{\left(P_{z0}-S_{iz}\right)}^{2}}$; Equation (6) is the Taylor expansion of the DS positioning equation at the predicted position $P_{0}$; h.o.t is the higher-order term of the Taylor expansion. For Equation (6), assuming that the higher-order terms are not considered, there are three unknowns: $P_{x0},P_{y0},P_{z0}$. Thus, neglecting temporal and other inaccuracies, observing the satellite at three distinct times suffices to determine the user’s position.

To facilitate comprehension and computation, we recast Equation (6) into state space form, as follows:

(7) $$f_{d}=f_{d0}+A(P-P_{0}),$$

where $f_{d}={\left(f_{d1},f_{d2},f_{d3}\right)}^{T}$, $f_{d0}={\left(f_{d10},f_{d20},f_{d30}\right)}^{T}$. The state transition matrix A is as follows:

(8) $$A=\frac{f}{c}\left[\begin{array}{ccc} a_{x1} & a_{y1} & a_{z1}\\ a_{x2} & a_{y2} & a_{z2}\\ a_{x3} & a_{y3} & a_{z3} \end{array}\right],$$

where $a_{xi}=\frac{V_{six}}{d_{i}}-\frac{V_{si}{\left(P_{0}-S_{i}\right)}^{T}(P_{x0}-S_{ix})}{d_{i}^{3}}$, $a_{yi}=\frac{V_{siy}}{d_{i}}-\frac{V_{si}{\left(P_{0}-S_{i}\right)}^{T}(P_{y0}-S_{iy})}{d_{i}^{3}}$, $a_{zi}=\frac{V_{siz}}{d_{i}}-\frac{V_{si}{\left(P_{0}-S_{i}\right)}^{T}(P_{z0}-S_{iz})}{d_{i}^{3}}$.

### 2.3. PDOP

The estimated value of DS can be computed at the forecasted position P_0_. Based on Equation (7), we derive the following conclusion:

(9) $$\Delta f_{d}=f_{d}-{\hat{f}}_{d}=A\Delta r+\varepsilon ,$$

where $\Delta r={\left(P-P_{0}\right)}^{T}$ is the difference between the actual value of the receiver position and the predicted value. Ignoring the higher-order error vector ε, from the least squares method, it can be inferred that

(10) $$\Delta r={\left(A^{T}A\right)}^{-1}A^{T}\Delta f_{d},$$

Referring to the definition of PDOP in GNSS positioning, according to the above Equation (10), the PDOP in the doppler shift positioning of communication satellite SOPs can be defined as follows:

(11) $$\mathrm{PDOP}=\sqrt{tr{\left(A^{T}A\right)}^{-1}},$$

According to the existing studies, we can see that, the smaller the PDOP, the better the positioning accuracy.

## 3. The Influence of Satellite Elevation Angle on Positioning Accuracy

**Figure 3:**
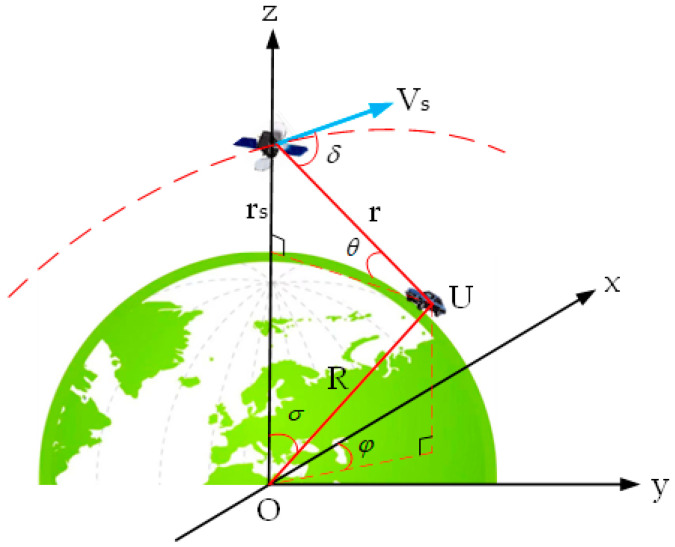
The place schematic diagram of user U and satellite.

For the convenience of analysis and calculation, this article mainly refers to the single satellite error amplification factor in the literature [27] to study the impact of the elevation of low orbit communication satellites relative to users on positioning accuracy as follows:

(12) $$\left|\frac{r}{V_{s}\cdot \sin\delta }\right|=\frac{R^{2}+{\left(r_{s}+R\right)}^{2}-2\cdot R\cdot (r_{s}+R)\cdot \cos\sigma }{{\left[\frac{\mu }{\left(r_{s}+R\right)}\cdot \left(R^{2}+{\left(r_{s}+R\right)}^{2}-2\cdot R\cdot (r_{s}+R)\cdot \cos\sigma -R^{2}\cdot \sin^{2}\sigma \cdot \cos^{2}\varphi \right)\right]}^{\frac{1}{2}}},$$

where $\mu =3.986005\times 10^{14}$ is the gravitational constant, $R=6.368\times 10^{6}$ is the Earth radius, $V_{s}=(\sqrt{\frac{\mu }{r_{s}+R}},0,0)$ is the satellite vector velocity, U=(Rsinσcosφ,Rsinσsinφ,Rcosσ) is the position of the user, $r_{s}$ is the satellite orbital altitude, and the definitions of σ and φ are shown in Figure 3.

From Figure 3, it can be seen that, after the satellite is determined, the change in σ reflects the change in φ; that is, the smaller the σ, the larger the φ. Therefore, the impact of satellite elevation θ on positioning accuracy can be obtained by analyzing the impact of changes in σ on the positioning accuracy. The larger the elevation θ, the better the positioning accuracy.

**Figure 4:**
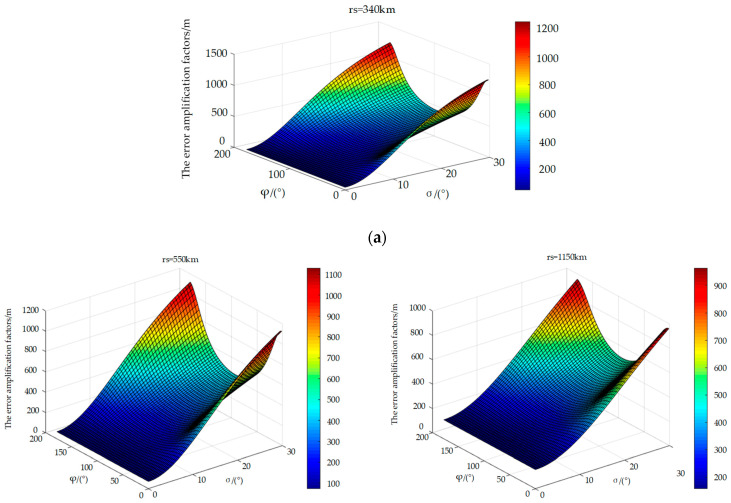
The error amplification factors of a single satellite at different satellite orbital altitudes. (**a**) The error amplification factors of a single satellite at the satellite orbital altitude of 340 km. (**b**) The error amplification factors of a single satellite at the satellite orbital altitude of 550 km. (**c**) The error amplification factors of a single satellite at the satellite orbital altitude of 1150 km.

Figure 4 show the error amplification factors of a single satellite at satellite orbital altitudes of 340 km, 550 km, and 1150 km, respectively. Through comparative analysis of the three images, it can be seen that, as the satellite orbit height increases, the maximum error also decreases.

As previously noted, the greater the satellite elevation angle, the lower the positioning error. The computation of the satellite elevation angle proceeds as follows:

(13) $$e_{su,ECEF}=\frac{S_{i}-P}{\left\|S_{i}\right.-\left.P\right\|},$$

(14) $$e_{su,ENU}=M\cdot e_{su,ECEF}={\left[e_{E}\;e_{N}\;e_{U}\right]}^{T},$$

(15) $$M=\left[\begin{array}{ccc} -\sin L & \cos L & 0\\ -\sin B\cos L & -\sin B\sin L & \cos B\\ \cos B\cos L & \cos B\sin L & \sin B \end{array}\right],$$

(16) $$El=\arctan\left(e_{U}\right),$$

where $e_{su,ECEF}$ is the unit vector of a satellite and user sight line direction in the Earth-centered Earth-fixed (ECEF) coordinate system. $e_{su,ENU}$ is the unit vector of a satellite and user sight line direction in the east–north–up (ENU) coordinate system. El is the elevation angle of the satellite relative to the user.

## 4. The Satellite Selection Strategy

**Figure 5:**
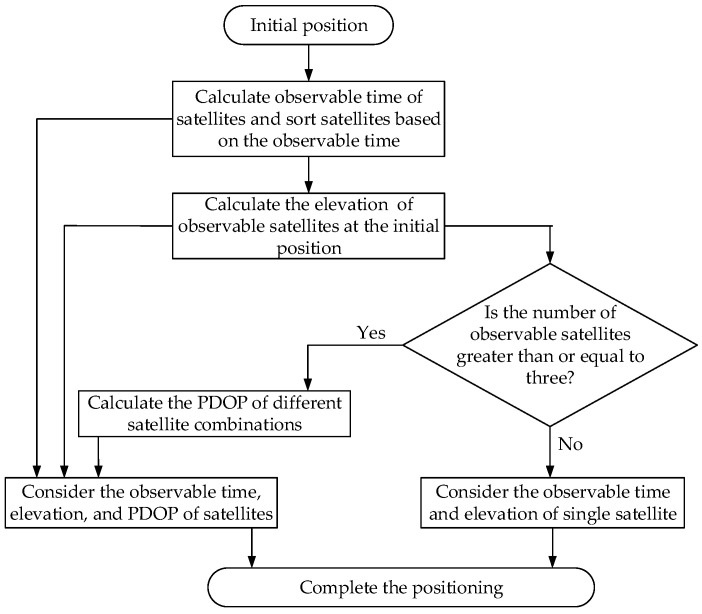
The flowchart of the satellite selection strategy.

Figure 5 illustrates the satellite selection strategy derived from the above analysis, taking into account the effects of satellite observable time, elevation, and PDOP on positioning accuracy. Initially, the satellite observable time at the current location can be forecasted using two-line orbital element (TLE), and satellites are ranked according to their observation times. Next, based on Equations (13)–(16) presented in Section 4, the elevation of the observable satellites is calculated. Lastly, the satellite PDOP at the initial position is computed, and the optimal satellite combination is selected to facilitate positioning.

## 5. Results and Discussion

### 5.1. Satellite Observable Time

**Figure 6:**
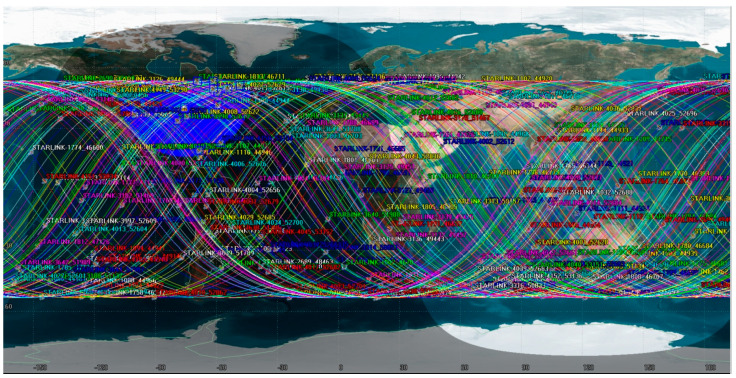
The satellite ground trace.

**Figure 7:**
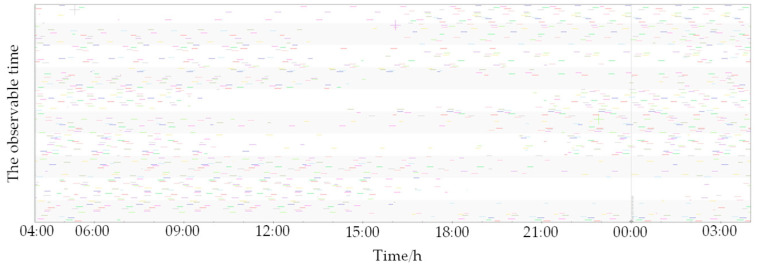
The observable time of 200 satellites in one day.

**Figure 8:**
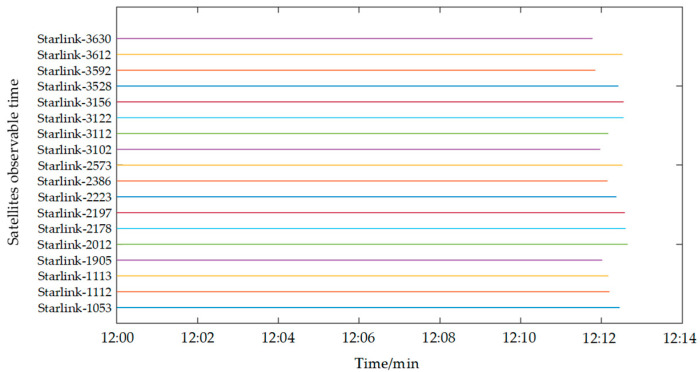
Satellite observable time.

This study samples 200 satellites from the existing Starlink constellation and computes their velocity and positional data using publicly accessible Two-Line Element (TLE) sets. Figure 6 illustrates the ground tracks of these satellites. An observation site was established in Xi’an, China, to monitor the selected 200 satellites. The visibility durations are depicted in Figure 7. Figure 7 reveals that the observation site maintains continuous visibility of at least one satellite at any given time, suggesting the practicality of SOPs positioning utilizing Starlink satellite. Following the doppler shift positioning principle, the user’s location can be ascertained. We chose 12:00 p.m. as the observation time, and Figure 8 indicates that 18 satellites are visible at this time, with their visibility periods fulfilling the positioning criteria. Figure 7 merely displays the visibility times of the 200 satellites, yet the positioning approach outlined in this paper essentially satisfies the positioning needs at all times. It is conceivable that, once the full deployment of both Starlink and China Satellite Network’s constellations is complete, a greater number of satellites will be available for positioning purposes.

### 5.2. Position Error and PDOP of a Single Satellite

**Figure 9:**
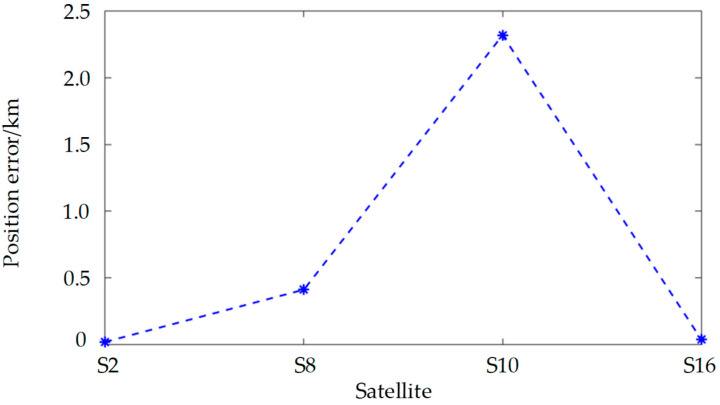
Single satellite position error.

As previously noted, the observation point is capable of simultaneously detecting 18 satellites. Nonetheless, for the purposes of this analysis, it is posited that only one of these 18 satellites is observable at any given moment. Consequently, the four satellites exhibiting the highest elevation angles are chosen for positioning. These satellites, in descending order of elevation, are identified as S2, S16, S8, and S10. Figure 9 illustrates the positioning errors associated with these four satellites over the duration of the observation period. The data indicate that satellite S2 incurs the least positioning error, whereas satellite S10 experiences the greatest error. This finding aligns with the conclusions drawn in the third section of this study. It is thus recommended that, when selecting a satellite for positioning, preference be given to those with higher elevation angles.

**Figure 10:**
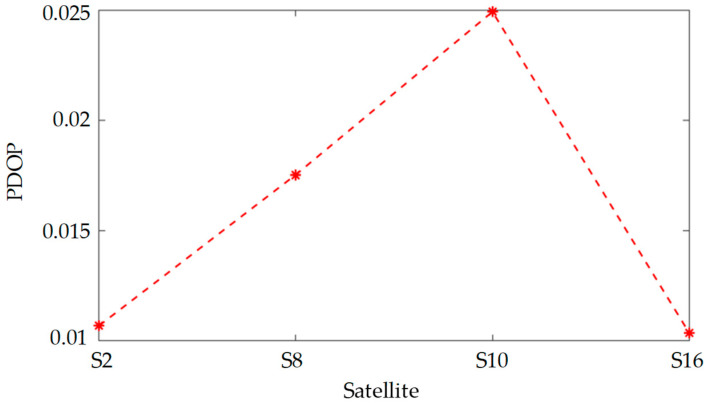
The PDOP of s single satellite.

Figure 10 presents the PDOP for a single satellite, which quantifies the variability in the satellite’s spatial orientation. A higher degree of variability corresponds to a lower PDOP value, signifying improved positioning precision. In Figure 10, it is evident that the PDOP values for satellites S2 and S16 are the lowest, suggesting that these satellites exhibit the most substantial positional fluctuations within the observable timeframe. As depicted in Figure 9, the positioning precision of satellites S2 and S16 surpasses that of satellites S8 and S10. Consequently, when observing a single satellite, it is advisable to select one that demonstrates significant positional changes during the observation period to achieve more accurate positioning.

### 5.3. Position Error and PDOP of the Satellite Permutations

**Figure 11:**
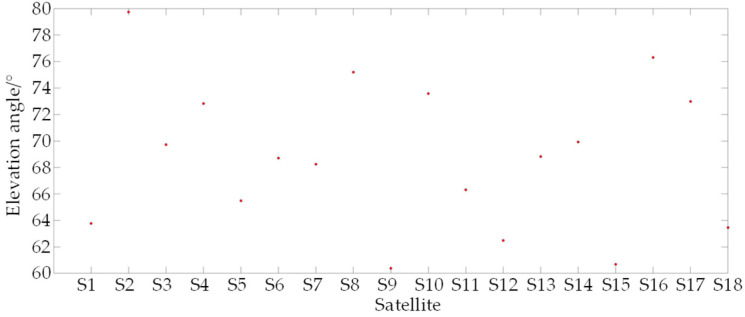
Satellite elevation angle.

**Figure 12:**
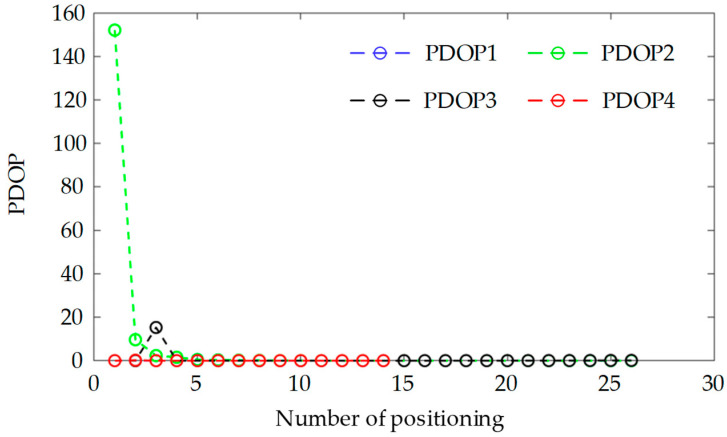
PDOP of the satellite permutations.

**Figure 13:**
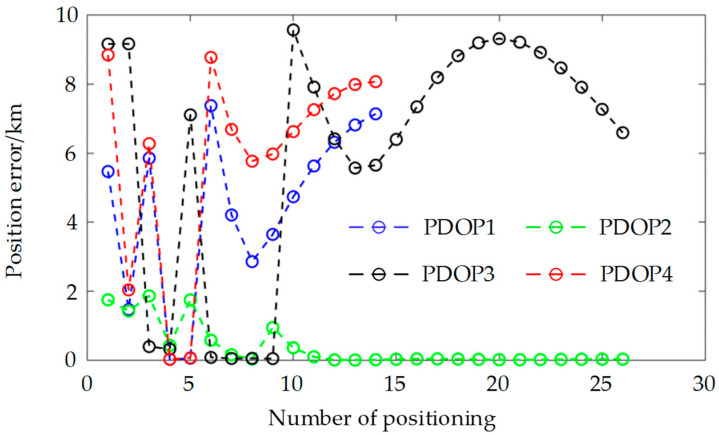
Position error of the satellite permutations.

For the sake of analysis simplicity, this paper disregards the influence of time on positioning accuracy, necessitating only three satellites to position the observation points. Consequently, adhering to the satellite selection criteria outlined in Section 4, when the number of observable satellites exceeds three, satellites with higher elevation angles and combinations with lower PDOP values should be chosen to achieve positioning. To minimize the computational burden, the four satellites with the highest elevation angles: S2, S16, S8, and S10 were selected from Figure 11, and the PDOP values for the permutations S2-S8-S10, S2-S8-S16, S2-S10-S16, and S8-S10-S16 were computed. Figure 12 displays PDOP1, PDOP2, PDOP3, and PDOP4, respectively. In Figure 12, it is apparent that the PDOP values of these satellites converge over the observable time period. Figure 13 illustrates the three-dimensional positioning errors for the aforementioned four satellite permutations. The positioning errors for the permutations S2-S8-S10, S2-S8-S16, S2-S10-S16, and S8-S10-S16 are denoted as Deltar1, Deltar2, Deltar3, and Deltar4, respectively. Figure 13 reveals that, over time, the positioning error Deltar2 for the permutation S2-S8-S16 stabilizes within 20 m, indicating superior positioning accuracy. Additionally, the elevation angles of the three satellites S2, S8, and S16 are the highest among the 18 satellites.

## 6. Conclusions

This manuscript analyzes and elaborates on the principle of doppler shift positioning for SOPs in LEO communication satellites. It derives and calculates the PDOP for the proposed doppler shift positioning method, drawing on the PDOP concept from GNSS. Due to the asynchronous nature of LEO communication satellites, the relative positions of satellites to users change in real time. Therefore, we have extended the PDOP concept to “single satellite PDOP”, which reflects the degree of satellite position change. In conclusion, the following points can be made:

(1) When there are less than three observable satellites, according to the impact of a single satellite on positioning accuracy in the third part of the article, the observable time and elevation angle of the satellite should be comprehensively considered to select the satellite for positioning.

(2) When more than three satellites are visible, it is crucial to consider not only those with high elevation angles and significant changes in orbital position but also the PDOP values of various satellite permutations.

Furthermore, the impact of the algorithm proposed in this paper on the time to first estimation is noteworthy. The algorithm calculates the position and velocity information of satellites based on the TLE of the satellite and completes satellite selection on the computing platform prior to positioning. Based on the current simulation results, the satellite selection algorithm does not significantly affect the first localization estimation. For instance, with Starlink, the observable time of satellites is currently on the order of minutes, whereas the processing time of the algorithm proposed in this paper is on the level of seconds. We will explore the impact of the algorithm on the time to first estimation in future research.

## Data Availability

The original data presented in the study are openly available at [http://celestrak.org/NORAD/elements/index.php?FORMAT=tle], accessed on 1 January 2025.
